# Fears in preschoolers: translation, reliability, and validity of the Fear Survey Schedule for Infant-Preschoolers (FSSIP) - Brazilian version

**DOI:** 10.47626/2237-6089-2020-0170

**Published:** 2021-03-31

**Authors:** Marina da Rocha, Thomas Ollendick, Felipe Alckmin-Carvalho, Livia Campos, Fernando Asbahr, Renatha El Rafihi-Ferreira

**Affiliations:** 1 Universidade Presbiteriana Mackenzie São Paulo SP Brazil Universidade Presbiteriana Mackenzie, São Paulo, SP, Brazil.; 2 Child Study Center Department of Psychology Virginia Tech Blacksburg VA USA Child Study Center, Department of Psychology, Virginia Tech, Blacksburg, VA, USA.; 3 Escola de Enfermagem Universidade de São Paulo São Paulo SP Brazil Escola de Enfermagem, Universidade de São Paulo, São Paulo, SP, Brazil.; 4 Instituto de Psiquiatria Hospital das Clínicas Faculdade de Medicina de São Paulo São Paulo SP Brazil Instituto de Psiquiatria, Hospital das Clínicas da Faculdade de Medicina de São Paulo, São Paulo, SP, Brazil.

**Keywords:** Preschool children, fear, child development, validation study

## Abstract

**Objective:**

To translate the Fear Survey Schedule for Infants-Preschoolers (FSSIP) into Brazilian Portuguese and to examine its reliability and validity for assessing fears among Brazilian preschoolers.

**Methods:**

Two independent bilingual professionals conducted translation and back-translation of the original survey. The translated version was used to assess 152 preschool children divided in two groups: Clinical - 71 children referred for treatment for nighttime fear, and Control - 81 children enrolled at kindergarten who had not been referred for any mental health service in the previous 6 months. All parents filled out the FSSIP, the Child Behavior Checklist (CBCL/1.5-5), and a sociodemographic questionnaire.

**Results:**

Cronbach’s alpha coefficients were 0.949 (95% CI: 0.94-0.96) for the entire sample; 0.948 (95% CI: 0.93-0.96) for the Clinical Group, and 0.95 (95% CI: 0.93-0.96) for the Control Group. The mean score for fears was higher in the clinical group (60.19 vs. 51.53, t = -2.056; p = 0.042), indicating acceptable discriminate validity. We also found positive, moderate, and statistically significant correlations between FSSIP and most CBCL scores, indicating good convergent validity.

**Conclusion:**

The Brazilian Portuguese version of the FSSIP showed good psychometric properties, and hence may be used in research and clinical settings to evaluate fears in preschoolers.

## Introduction

Fear is part of normal development and comprises a basic human emotion. It is an inborn emotional reaction produced by the perception of present or impending danger, leading to avoidance of threat, thereby having clear survival value.^1-3^ As part of normal development, child fears have been extensively investigated.^4-7^ However, for many individuals, fear is a significant problem and may impair normal development.^7,8^ Research indicates associations between fears in early childhood and the development of specific phobias and other anxiety disorders in early adolescence and adulthood.^9,10^ Therefore, development of instruments to assess fear in the pediatric age group is important and useful for clinical and scientific applications.

The Fear Survey Schedule for Children-Revised (FSSC-R)^11^ is an 80-item self-report questionnaire frequently used to assess fears in 7-to-18-year-old children and adolescents. It allows diverse fears to be investigated in boys and girls, encompassing different cultural backgrounds, and provides useful information on the relationships between fears and anxiety disorders. There is considerable evidence of its validity in clinical and community samples from various countries.^2,12-17^

Based on the FSSC-R,^11^ the Fear Survey Schedule for Infants-Preschoolers (FSSIP) was developed by Warren, Ollendick, and Simmens^18^ to assess fears in children from 18 months to 5 years of age (mean = 26 months, standard deviation [SD] = 9 months, interquartile range: 23-27 months). This measure differs from the FSSC-R because it includes 29 additional items pertinent to the lives of infant, toddlers and preschoolers (e.g., “clowns,” “having to go to daycare or preschool”) and excludes 17 items that are less relevant for children under 5 years old (e.g., “terrorists,” “taking a test”). The FSSIP is composed of 92 items referring to content of young children’s fears, for which parents respond on a scale ranging from 0 (none) to 2 (a lot). The higher the score, the greater the intensity of the fears. The Fear Score (FS) is the sum of the ratings based on all items and the High Intensity Fear Score (HIFS) is the total number of fears rated “a lot.”

The FSSIP was tested for its reliability and validity in the United States by Warren, Ollendick, and Simmens.^18^ Several methods were used to examine its convergent validity. Parents and other caregivers were interviewed and completed questionnaires concerning children’s fears and child fearful behavior was directly observed in a structured play setting. Measures (Diagnostic Interview for Infants to Preschoolers for Anxiety, Infant Toddler Social and Emotional Assessment, Child Behavior Checklist, and Caregiver-Teacher Report Form) used to test convergent validity were significantly correlated with the FS and HIFS. The authors found both scoring methods showed good internal consistency, low-to-moderately high test-retest correlations and good convergent and discriminant validity for an English-speaking sample.^18^ The two scoring methods correlated highly, and there was no compelling evidence that one was better than the other.

The different versions of the FSSC-R for children and adolescents^2,11^ and infants and preschoolers^18^ have been used in studies with international samples and are considered evidence-based instruments for identification and evaluation of children’s fears.^2,12-17,19,20^

The presence of frequent and intense fears in preschool children is related to difficulties in developing autonomy, independence, and other socio-emotional skills necessary in adolescence and adulthood. In addition, if not identified and treated early, childhood fears may evolve into specific phobias and/or other anxiety disorders, conditions associated with impairments in adaptive behavior and socioemotional functioning.^8,9,21^

Thus, early identification and treatment of fears in preschool children constitute low-cost interventions that may prevent mental health problems in the future. However, to date, no instruments for assessing fears in preschoolers have been developed in Brazil and no studies reporting the translation and validation of international instruments could be found. The aim of this study was to translate the FSSIP into Brazilian Portuguese and to examine its reliability and validity for assessing fears among Brazilian preschoolers.

## Methods

The study was carried out in two phases. First, translation and back-translation were performed. After that, the new Brazilian Portuguese version of the FSSIP was filled out by the parents of two groups of children: the first comprised children referred for treatment due to nighttime fears and the second was a control group (non-referred children).

### Phase 1

The Brazilian version of the FSSIP was prepared in two steps. First, two bilingual professionals translated the questionnaire into Brazilian Portuguese independently. In the second step, the two translations were randomly distributed to two bilingual professionals. Also independently, these two professionals back-translated the two Portuguese translations developed in the first step back into English. The most accurate translation was chosen by consensus, considering conceptual and semantic equivalence and cultural appropriateness.

### Phase 2

Parents of children aged between 2 and 6 years were recruited from two sources to select a mix of anxious and non-anxious children: (1) a kindergarten; and (2) a psychology school clinic that offered treatment for nighttime fears. After conclusion of the translation process, the Brazilian version of the FSSIP was used in a randomized clinical trial that evaluated the effectiveness of a bibliotherapy treatment program for nighttime fears in young children^22^ and a second cross-sectional study aimed at assessing emotional/behavioral problems in preschool children not referred for mental health services.^23^

### Participants

A total of 152 parents of children of both genders aged 2–6 years (M = 4.30, SD = 1.09) participated in the study. This was a non-probabilistic sample, selected by convenience criteria. Participants were divided into two groups:

- Group 1: 71 children (M = 4.96, SD = 0.90) referred for clinical treatment who met criteria for a diagnosis of separation anxiety or specific phobia of the dark according to the Diagnostic and Statistical Manual of Mental Health Disorders, Fifth Edition (DSM-5).^24^
- Group 2: 81 children (M =3.73, SD = 0.89), enrolled at a kindergarten in the city of São Paulo. The children’s parents declared that they had not been referred to a mental health service in the previous 6 months.

For both groups, children with neurological impairment and/or psychotic symptoms reported by parents were excluded.

All participants provided written consent. Both studies were approved by ethical committees (Approval numbers: 2.541.684 and 1.505.273). All ethical guidelines for research with human beings were followed.

### Measures

#### Sociodemographic questionnaire

Elaborated by the researchers to gather information regarding: age, gender, parental educational level, marital status, and social status of the family.

#### Fear Survey Schedule for Infant-Preschoolers (FSSIP)

Developed and validated in the United States,^18^ this instrument is for screening and identifying the content of fears of children aged 18 months to 5 years old, considering the parental perspective. The 92 items have a Likert response scale on which 0 means absence of fear; 1, some fear; and 2 a lot of fear. The higher the score, the greater the intensity of the fears. For each child, two scores are calculated: a) FS - based on the sum of the scores of all items (mean score was 24.3 [range = 0 – 99] in the US sample), and b) the HIFS - a score which equates to the number of fears that were rated “a lot”, for which the authors reported a mean score of 3.3 (range = 0 - 29).^18^ In the original study, the FSSIP demonstrated high internal consistency, acceptable test-retest correlations over an average of 6 months, and good convergent and discriminant validity.^18^

#### Child Behavior Checklist for ages 1.5 to 5 (CBCL/1.5-5)

Developed by Achenbach & Rescorla^25^ to obtain standardized ratings of behavioral and emotional problems in children aged 18 months to 5 years of age, based on parents’ reports. The 99 items are rated on a 3-point Likert scale on which 0 means “Not True – as far as you know”, 1 means “Somewhat or Sometimes True”, and 2 means “Very True or Often True”. The items are grouped into 7 empirically-based scales: Emotional Reactive, Anxiety/Depression, Somatic Complaints, Withdrawal, Sleep Problems, Attention Problems, and Aggressive Behavior. These scales form three broad-band scales: Internalizing (the sum of the 4 first scales), Externalizing (the sum of the last 2 scales), and Total Problems (which includes all problem items). The Sleep Problems scale scores are included in the Total Problems Scale but not the Internalizing or Externalizing Problems Scales. Besides the empirically-based scales, the CBCL also groups items in five DSM-oriented scales: Affective Problems, Anxiety Problems, Autism Spectrum Problems, Attention Deficit/Hyperactivity Problems, and Oppositional Defiant Problems. Scores for both empirically-based and DSM scales are calculated based on the sum of the items that are included in the scale and may be classified as normal, borderline or clinical range in comparison to the normative sample. For the present study, borderline and clinical ranges are grouped together to avoid false negatives, as suggested by Achenbach & Rescorla.^25^

The Brazilian version of the CBCL/1.5–5 possesses good reliability indexes: test-retest analysis interclass correlation coefficients of 0.99 for internalizing problems, 0.99 for externalizing problems and 0.98 for total problems.^26^ It also presents acceptable internal consistency values (Cronbach’s alpha) ranging from 0.69 (somatic problems) to 0.94 (total problems)^26^; and good sensitivity discriminating children with Autism Spectrum Disorder (ASD) from children with neurotypical development and children with Attention Deficit Hyperactivity Disorder (ADHD).^27^

#### Statistical analysis

Descriptive analyses were conducted for the sociodemographic data, and for the scores on both questionnaires (FSSIP and CBCL). The internal consistency of the FSSIP was calculated using Cronbach’s alpha and its respective confidence interval was obtained using non-parametric bootstrap measures for the 2.5 and 97.5 percentiles. Spearman’s correlation coefficients were used to generate item-total correlations for the FSSIP, as well as the correlation between the FSSIP and CBCL scales. After verifying the assumption of normal distribution, FSSIP scores were compared between groups 1 (clinical) and 2 (control) using student’s *t *test for independent measures. All hypothesis tests used were two-tailed.

## Results

### Phase 1

The final version of the Brazilian Portuguese FSSIP was obtained after completion of the translation and cultural adaptation procedures. It is presented in Table 1.

**Table 1:** Brazilian Portuguese Translation of FSSIP items

Brazilian Portuguese Translation of FSSIP items		
1. Barulhos altos	32. Armas	63. Ter que vestir roupas diferentes dos outros
2. Andar de carro ou de ônibus	33. Estar em uma briga	64. Ser punido
3. Ser repreendido pelo pai ou mãe	34. Fogo – se queimar	65. Mãos descuidadas ou sujas
4. Lagartos	35. Cortar-se ou machucar-se	66. Errar
5. Ter que se apresentar na frente de outras pessoas (música, dança, etc.)	36. Estar no meio de uma multidão	67. Filmes assustadores
6. Fantasmas ou coisas assustadoras	37. Trovoadas	68. Som de sirenes
7. Objetos afiados	38. Ter que comer um novo alimento	69. Fazer algo novo
8. Ter que ir para o hospital	39. Gatos	70. Germes ou ter uma doença grave
9. Morte ou pessoas mortas	40. Pessoas usando máscaras	71. Espaços fechados
10. Se perder em um lugar estranho	41. Ser atropelado por um carro ou caminhão	72. Terremotos
11. Cobras	42. Ter de ir para a creche ou pré-escola	73. Ser corrigido pelo pai ou mãe
12. Falar ao telefone	43. Crianças brincando de forma violenta	74. Elevadores
13. Montanha-russa ou brinquedos de parques de diversão (em parques de diversão)	44. Pais discutindo	75. Lugares escuros
14. Aspirador de pó	45. Quartos ou armários escuros	76. Não conseguir respirar
15. Receber reconhecimento (elogios ou prêmios) na frente dos outros	46. Marionetes ou Teatro Infantil	77. Ser picado por abelha
16. Andar de trem	47. Formigas ou besouros	78. Minhocas ou caracóis
17. Ficar em casa com uma babá	48. Pessoas fantasiadas	79. Ratos ou camundongos
18. Ursos ou lobos	49. Pessoas de aparência estranha	80. Mudança na rotina
19. Encontrar alguém pela primeira vez	50. Sangue	81. Brinquedos ou coisas fora do lugar
20. Bombardeios – ser invadido	51. Ir ao médico	82. Sujeira
21. Tomar injeção aplicada por enfermeira ou médico	52. Cachorros de aparência estranha ou má	83. Monstros
22. Ir ao dentista	53. Cemitérios	84. Algumas lojas
23. Lugares altos como montanhas ou edifícios altos	54. Cabelo desarrumado	85. Ter que dormir longe do pai ou da mãe
24. Ser provocado	55. Cortar o cabelo	86. Piscina
25. Aranhas	56. Águas profundas ou oceano	87. Banheiro
26. Um ladrão invadir sua casa	57. Pesadelos	88. Ter que visitar parentes
27. Andar de avião	58. Cair de lugares altos	89. Andar rápido (em um carro, carrinho de criança, etc.)
28. Palhaços	59. Tomar um choque elétrico	90. Tomar banho
29. Ter que falar com um estranho	60. Ir para a cama no escuro	91. Balanços
30. Morcegos ou pássaros	61. Enjoar no carro	92. Ter que sair de casa
31. Separar-se do pai ou da mãe	62. Ficar sozinho(a)	

### Phase 2

The FSSIP – Brazilian Portuguese version was used to assess two groups of children: a group referred for psychological treatment and a control group (not referred for any mental health service in the previous six-months). Overall, the sample presented a balanced proportion considering sex and age, with more girls in both groups, and a mean age of 4 years. Approximately three out of four parents were married, and most of them had at least a high school level diploma. However, it is important to note that the frequency of parents with university education was higher in the clinical group and the median family income was also higher in this group. Table 2 contains the descriptive analysis of the sample’s sociodemographic characteristics.

**Table 2:** Description of the sample’s sociodemographic characteristics

	Total (n = 152)	Control (n = 81)	Clinical (n = 71)	p-value
Gender, n (%)				
Female	86 (56.6)	48 (59.3)	38 (53.5)	0.115*
Male	66 (43.4)	33 (40.7)	33 (46.5)	
				
Age (years)				
Mean±SD	4.3±1.1	3.7 ±0.9	4.9±0.9	0.0001†
				
Marital status, n (%)				
Separated	36 (23.7)	23 (28.4)	13 (18.3)	0.145*
Married	116 (76.3)	58 (71.6)	58 (81.7)	
				
Educational Level, n (%)				
Elementary education	21 (13.8)	20 (24.7)	1 (4.8)	0.0001*
Secondary education	60 (39.5)	43 (53.1)	17 (23.9)	
University level	71 (46.7)	18 (22.2)	53 (74.6)	
				
Family income (R$)^‡^				
Median	2,800.00	2,500.00	4,000.00	0.002^†^

SD = standard deviation.

* Chi-square test.

^†^*t* test for independent samples.

^‡^ Missing values (n = 11).

Considering that the original FSSIP has a single factor structure, the internal consistency of the FSSIP – Brazilian Portuguese was calculated considering all items. Cronbach’s alpha coefficients were 0.949 (95%CI: 0.94-0.96) for the total sample, 0.948 (95%CI: 0.93-0.96) for the control group, and 0.950 (95%CI: 0.93-0.96) for the clinical group. The alpha coefficient was not improved by exclusion of any of the items. The mean item-total correlation was 0.394 (ranging from 0.062 - Item 12 - to 0.661 – item 26). These indices are remarkably similar to those reported by Warren and colleagues.^18^

To verify the FSSIP’s discriminative capacity, the mean total scores obtained by the children in both groups were compared. Mean differences were significant: non-referred children achieved lower scores in comparison with the clinical group (51.53 [SD = 26.05] vs. 60.19 [SD = 25.77], t = -2.056; p = 0.042). Similar results were found when high-intensity fear scores were compared: the clinical group’s mean score was 32.39 (SD = 21.99), while the control group achieved 25.41 (21.67) (t = -1,970; p = 0.051). In the original study conducted in the US, the mean score was 24.3 and the mean HIFS score was 3.3, suggesting that total fear scores as well as the high intensity fear scores were considerably higher in both Brazilian samples than they were in the US. The range of scores for the Brazilian sample was also wider than what was reported for the original USA sample (0 to 99): control group scores ranged from 1 to 114, while clinical group scores ranged from 17 to 110. Moreover, the HIFS ranged from 0 to 88 (control group) and from 0 to 82 (clinical group).

Table 3 shows Spearman coefficients for correlations between the total FSSIP score and the CBCL emotional/behavioral problem scales, for the total sample and for the control and clinical groups separately. Results indicate correlations that varied from weak to moderate intensity,^28^ most of which were significant.

**Table 3:** Spearman coefficients for correlations between FSSIP total score and CBCL stratified by group

CBCL	Total (n = 152)	Control (n = 81)	Clinical (n = 71)
Emotionally reactive	0.401*^‡^	0.346*^‡^	0.424*^†^
Anxiety/depression	0.402*^‡^	0.350*^‡^	0.422*^†^
Somatic complaints	0.103^§^	0.139^§^	0.061
Withdrawn	0.281*^§^	0.236*^§^	0.311*^‡^
Sleep problems	0.269*^§^	0.287*^§^	0.15^5^§
Attention problems	0.177*^§^	0.176^§^	0.106^§^
Aggressive behavior	0.292*^§^	0.306*^‡^	0.203^§^
			
Internalizing problems	0.393*^‡^	0.400*^†^	0.330*^‡^
Externalizing problems	0.281*^§^	0.280*^§^	0.198^§^
Total problems	0.400*^‡^	0.412*^†^	0.338*^‡^
			
DSM – Affective problems	0.330*^‡^	0.325*^‡^	0.272*^§^
DSM – Anxiety problems	0.438*^‡^	0.391*^‡^	0.522*^†^
DSM – Autism spectrum problems	0.411*^‡^	0.322*^‡^	0.474*^‡^
DSM – Attention deficit/hyperactivity problems	0.259*^§^	0.219*^§^	0.225^§^
DSM – Oppositional defiant problems	0.216*^§^	0.184^§^	0.153^§^

* p < 0.05.

^†^ Strong effect size.

^‡^ Moderate effect size.

^§^ Weak effect size.

## Discussion

The results of our study suggest that the Brazilian Portuguese version of the FSSIP is both reliable and valid for assessment of the fears of Brazilian preschool children. To our knowledge, this is the first study aimed at assessing fears in Portuguese-speaking children.

Parents had no difficulty in understanding the FSSIP items, which indicates good content validity. The instrument’s internal consistency was adequate for the total sample and for the control and clinical groups. Aside from the original study,^18^ we did not find any other validation studies of FSSIP for use in other countries. Thus, it was not possible to compare the internal consistency observed after administration of the instrument to the Brazilian sample to that of other countries. However, here, we showed that the internal consistencies for children in our Brazilian sample were similar to those observed for the children in the original US study.

Presence of fears is common among school children and preschoolers.^7,29^ However, for some boys and girls, the frequency and intensity of these fears may produce social and emotional impact.^7,21^ Several studies indicate that early detection and treatment of fears that impair adaptive behavior among preschool children is a preventive measure against mental disorders in the future.^7,8,10,21^

From their parents’ perspective, the Brazilian preschool children evaluated in this study had more fears than American children.^18^ In the present study, we found mean total FSSIP scores that were significantly higher than the mean reported in the American study, both in the clinical and control group. The number of feared situations, objects, or animals was also much higher. One should analyze this result with caution. It may indicate that this sample of Brazilian children is actually more fearful or that Brazilian children are more exposed to situations with the potential to produce fear, such as urban violence and dangerous animals. Finally, the higher mean fears scores found among Brazilian children, regardless of whether they had been referred to mental health services, could be due to cultural differences in the interpretation of fear and its intensity.

In our study, we found moderate, positive, and statistically significant correlations between the fear scores, assessed by the FSSIP, and the CBCL emotional reactivity, depression, and anxiety subscales in the clinical group. This result corroborates several studies in which children’s fears were associated with anxiety disorders.^7,8,10,21^ Therefore, identifying and treating fears among preschool children may be associated with better prognosis in relation to the emotional difficulties of the internalizing profile.

There are many methods to assess fears in preschool children, such as clinical interviews with parents and/or caregivers, observation of children’s behavior in interaction in different contexts, or use of standardized instruments that consider parents’ perceptions of their children’s development. This last method of assessing fears has the advantage of easy administration and low cost, since its administration does not require the presence of a mental health professional.

However, to date, no standardized instrument for which the psychometric properties have been evaluated had been available for this assessment in Brazil. Thus, validation of the FSSIP for Brazilian Portuguese fills an important gap in assessment of fears among Brazilian children. Based on its psychometric properties, the instrument appears useful for new research investigations and for mental health professionals’ clinical practice.

Despite this study’s strengths, there are also some limitations that must be addressed. Selection of a non-probabilistic sample reduces the internal validity of the study and contributes to type II error – not finding statistically significant differences or correlations when they do exist. Moreover, selection of participants based on convenience criteria may have yielded information about children’s fears that is not representative of the population studied. Significant differences were found between clinical and control groups in terms of age, formal education, and family income: families in the clinical group had higher income and more formal education than those from the control group (who were from low-income families living in a region of high social vulnerability); furthermore, the children in the clinical group were older. Additionally, since children from vulnerable families tend to have more fears and anxiety than those living in high-income areas,^30^ it is possible that the fear and anxiety scores in the control group are overestimated and influenced by this variable.

Future studies are warranted to estimate the temporal stability of FSSIP by test-retest reliability and to produce additional evidence of the instrument’s validity, as well as studies involving administration of the scale to probabilistic samples of children referred and not referred to health services because of fears and distributed in homogeneous groups in terms of sociodemographic variables.
